# Supplementary material for: Comparison of Nozzle-Based and Nozzle-Free Electrospinning for Preparation of Fast-Dissolving Nanofibers Loaded with Ciprofloxacin
Source: Pharmaceutics. 2022 Jul 27;14(8):1559. doi: 10.3390/pharmaceutics14081559 (PMC9413034; doi:10.3390/pharmaceutics14081559)
Supplement: Supplementary file 1 [file pharmaceutics-14-01559-s001.zip › pharmaceutics-1748025-supplementary.pdf]

Supplementary Material

Comparison of nozzle-based and nozzle-free electrospinning for preparation of fast-dissolving nanofibers loaded with ciprofloxacin

Raman spectral profiling

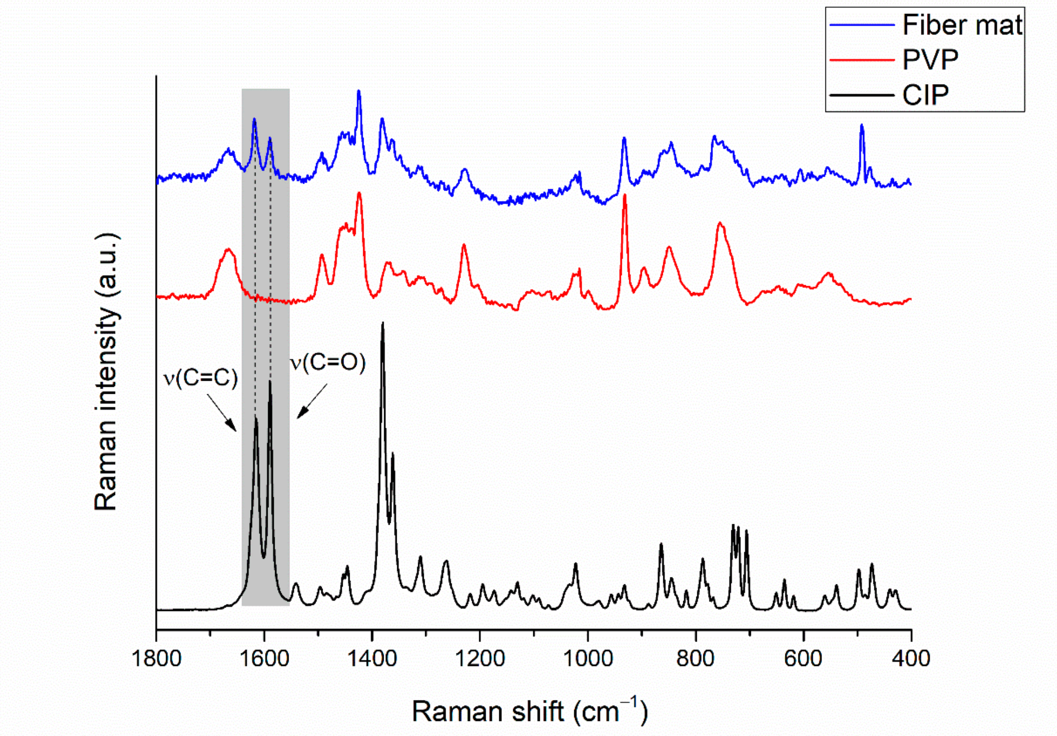

Figure S1. Raman spectra of investigated fiber mat and initial components, indicating the unique spectral range of CIP applied for constructing Raman chemical maps.

For investigating the distribution of CIP in the fiber mats the spectral range from 1650 to 1550 cm^{-1} was selected for profiling to determine the frequency of occurrence of CIP in the electrospun specimens. The selected spectral region contained two intense signals centered at 1615 cm^{-1} because of the aromatic ring stretching mode ν(C=C) of the quinolone ring system coupled with the carbonyl stretching mode ν(C=O) at 1589 cm^{-1} were suitable for analysis, as in this spectral region no overlapping band of PVP could be found.

Linearity of HPLC method

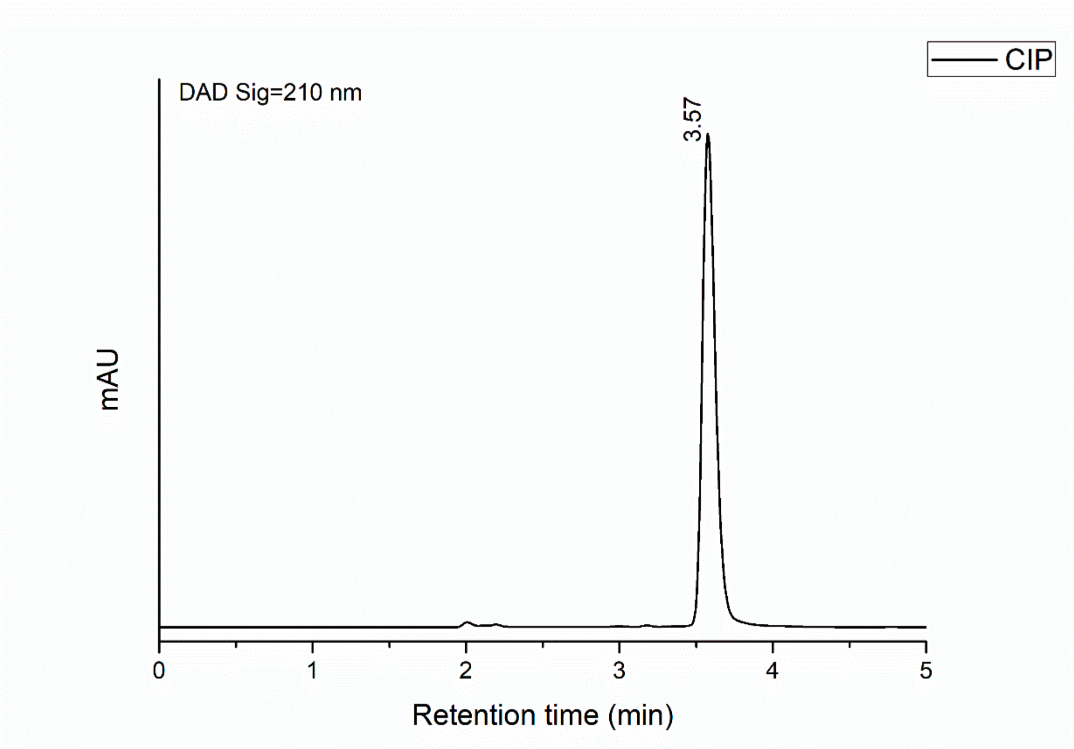

Figure S2. Chromatogram of CIP determined by the applied HPLC method.

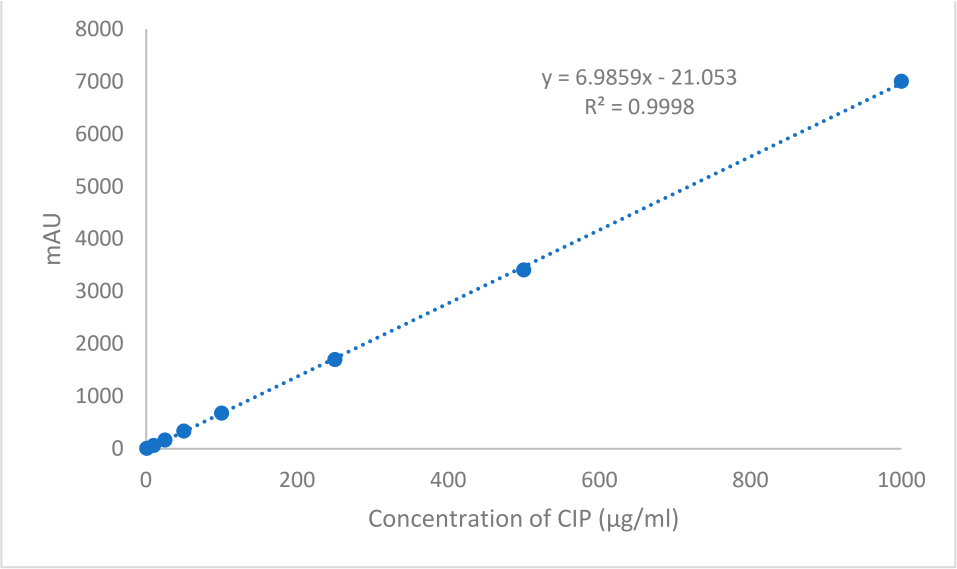

Figure S3. Linearity of CIP in the concentration range of 1–1000 µg/mL.
